# Honey vs. Mite—A Trade-Off Strategy by Applying Summer Brood Interruption for *Varroa destructor* Control in the Mediterranean Region

**DOI:** 10.3390/insects14090751

**Published:** 2023-09-07

**Authors:** Marin Kovačić, Aleksandar Uzunov, Ivana Tlak Gajger, Marco Pietropaoli, Victoria Soroker, Noureddine Adjlane, Valerija Benko, Leonidas Charistos, Raffaele Dall’Olio, Giovanni Formato, Fani Hatjina, Valeria Malagnini, Fabrizio Freda, Asaf Otmi, Zlatko Puškadija, Claudio Villar, Ralph Büchler

**Affiliations:** 1 Faculty of Agrobiotechnical Sciences Osijek, J.J. Strossmayer University of Osijek, V. Preloga 1, 31000 Osijek, Croatia; marin.kovacic@fazos.hr (M.K.); zpuskadi@fazos.hr (Z.P.); 2 Faculty of Agricultural Sciences and Food, Ss. Cyril and Methodius University in Skopje, 1000 Skopje, North Macedonia; uzunov@fznh.ukim.edu.mk; 3 State Key Laboratory of Resource Insects, Institute of Apicultural Research, Chinese Academy of Agricultural Sciences, Beijing 100193, China; 4 Department for Biology and Pathology of Fish and Bees, Faculty of Veterinary Medicine, University of Zagreb, Heinzelova ul. 55, 10000 Zagreb, Croatia; vbenko@vef.unizg.hr; 5 Istituto Zooprofilattico Sperimentale del Lazio e della Toscana “M. Aleandri”, Via Appia Nuova 1411, 00178 Rome, Italy; marco.pietropaoli@izslt.it (M.P.); giovanni.formato@izslt.it (G.F.); 6 Agricultural Research Organization (ARO), The Volcani Center, 68 HaMacabim Road, Rishon LeZion 7505101, Israel; sorokerv@volcani.agri.gov.il (V.S.); assafot@volcani.agri.gov.il (A.O.); 7 Department of Agronomy, Faculty of Science, University of Boumerdes, 35000 Boumerdes, Algeria; adjlanenoureddine@hotmail.com; 8 Department of Apiculture, Institute of Animal Sciences, Ellinikos Georgikos Organismos “DIMITRA”, 63 200 Nea Moudania, Greece; leoharistos@gmail.com (L.C.); fhatjina@gmail.com (F.H.); 9 BeeSources, 40132 Bologna, Italy; raffaele.dallolio@gmail.com; 10 Fondazione Edmund Mach, Centro Trasferimento Tecnologico, Via E. Mach, 1 San Michele all’Adige, 38098 Trento, Italy; valeria.malagnini@fmach.it (V.M.); fabriziofreda89@gmail.com (F.F.); 11 Consejería de Agricultura de la Junta de Comunidades de Castilla La Mancha, 02600 Albacete, Spain; claudiov@jccm.es; 12 Hintergasse 30, 35274 Kirchhain, Germany; ralph.buechler@t-online.de

**Keywords:** honey bee, *Varroa destructor*, queen caging, honey yield

## Abstract

**Simple Summary:**

Ectoparasitic mite *Varroa destructor* with its associated viruses is a common global threat to the health of honey bee colonies. If colonies are not treated, the vast majority die in a 3-year period. Existing acaricides used for treatment are becoming less effective, and new approaches to honey bee protection are required. A reliable method is to create a broodless condition in a colony by preventing the queen from laying eggs, and after 25 days all mites will be exposed to the treatment with organic acids or essential oils. The focus of our study, performed on 178 colonies in six Mediterranean countries, was to compare different periods of queen caging on honey production, colony development, and the effect of treatment. Queen caging had no negative effect on colony strength before the wintering period, while it affected honey production; colonies in which queens were caged two weeks before the main summer nectar flow produced significantly less honey. However, tested colonies ten weeks after the treatment had significantly lower infestation with *V. destructor* mites. This study shows that caging the queen with subsequent oxalic acid treatment 25 days after caging is an efficient method to control *V. destructor* infestation, while the starting point of queen caging in relation to the main summer nectar flow affects honey production.

**Abstract:**

In this study, we investigated the effect of queen caging on honey bee colonies’ post-treatment development and the optimal timing of method application on honey production during the main summer nectar flow. We conducted the study in nine apiaries (N = 9) across six Mediterranean countries, with a total of 178 colonies. The colonies were divided into three test groups: QC1, QC2, and C. The QC1 group involved queens caged for a total of 28 days before the expected harvesting day. In the QC2 group, queens were caged for 28 days, but only 14 days before the expected harvesting day. The C group consisted of queens that were not caged, and the colonies received common local treatments. In both the QC1 and QC2 groups, the colonies were treated with a 4.2% oxalic acid (OA) solution by trickling after the queen release. Our findings revealed no significant adverse effects (*p* > 0.05) on colony strength at the end of the study resulting from queen caging. However, significantly lower amounts of honey were extracted from the QC1 group compared to both the QC2 group (*p* = 0.001) and the C group (*p* = 0.009). Although there were no initial differences in *Varroa destructor* infestation between the groups, ten weeks later, a significantly higher infestation was detected in the C group compared to both the QC1 group (*p* < 0.01) and the QC2 group (*p* = 0.003). Overall, our study demonstrates that queen caging, in combination with the use of OA, is an effective treatment for controlling *V. destructor*. However, the timing of caging plays a crucial role in honey production outcomes.

## 1. Introduction

The *Varroa destructor* mite is an ectoparasite of the honey bee (*Apis mellifera* L.) and is recognized as the leading cause of worldwide colony losses [1,2]. From the beginning of the invasion, beekeepers prioritized using chemical substances, mainly synthetic acaricides [3]. Even over half a century later, synthetic chemicals are commonly used by many beekeeper operations despite the potential of residues in hive products [4,5] and, more importantly, *V. destructor* mite resistance due to overuse of these chemicals [6,7,8]. These aspects primarily threaten consumers’ safety and sustainable beekeeping management.

In parallel, alternative beekeeping techniques, known as api-biotechnical methods, were developed to counteract *V. destructor* with limited or no use of acaricides. A comprehensive overview of different api-biotechnical methods to prevent and control mite infestation is given in an article by Rosenkranz et al., 2010 [9]. Many of those methods in beekeeping, such as screened bottom boards, trapping of mites in worker or drone brood, and colony arrangement, prevent reinfestation. Methods relying on a brood interruption during the active beekeeping season, followed by oxalic acid treatment, are currently gaining popularity among the beekeeping and research communities [10,11]. The fundamental mechanism behind this approach is trapping and physical removal of the mites in the sealed brood and/or treating the exposed mites (known as the phoretic or dispersal stage [12,13]), during the broodless conditions in the colony. Thus, the methods of brood removal, queen caging, and trapping comb seem best suited to the various beekeeping practices, particularly for the geographical regions with prolonged brood rearing [13].

Among the available acaricides for *V. destructor* control, oxalic acid shows high efficacy [14,15,16,17,18,19], does not leave residues in beehive products [20,21], and does not lead to resistance phenomena [22]. However, to achieve a high acaricide efficacy, colonies should be in a broodless stage, which in temperate climates may naturally happen only for a short period during the winter or seldom in dry summer season. In the brood’s presence, oxalic acid’s efficacy is less than 50% [19,23,24].

Several studies have shown that summer brood interruption combined with a subsequent OA application, either via the trickle or sublimation method, is an effective strategy to reduce *V. destructor* infestation [10,11,16,25,26,27,28] and virus load [28,29,30]. Furthermore, no adverse effects on honey production early in the season [31], and colony strength before winter, were detected [32,33].

To create a broodless condition, beekeepers can confine the queen for a defined period [10,13]. By caging a queen for 21 to 25 days (depending on the presence of drone brood), the colony becomes broodless, forcing mites into the dispersal phase when they are susceptible to organic acid treatments, like oxalic acid. Büchler et al. [10] demonstrated high efficacy of the method when 4.2% oxalic acid was applied by trickling after the caging period of 25 days. Previous results of studies combining queen caging and oxalic acid treatment look promising, but it is important to consider the consequences of such a treatment on honey production and honey bee colony development.

Beekeepers from both hobby and commercial sectors are predominantly concerned about queen performance, colony development, and honey production. Therefore, our study investigated both the timing and effect of the queen caging method combined with an oxalic acid treatment on the post-treatment colony development and honey production during the main summer nectar flow.

## 2. Materials and Methods

**Figure 1:**
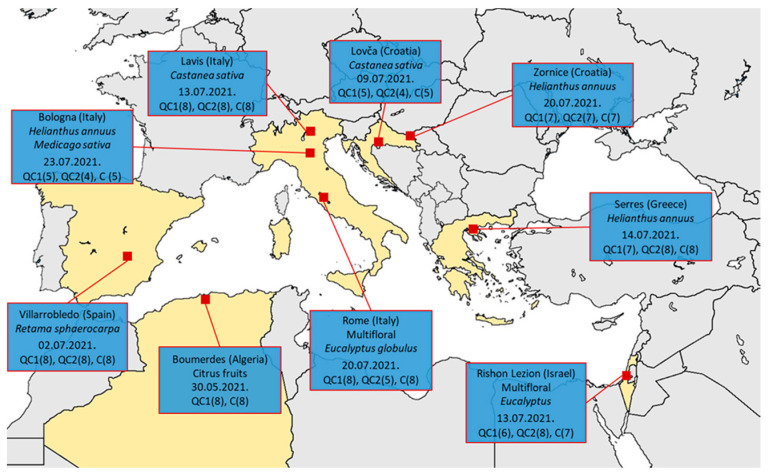
Map with test apiary’s location, main summer nectar sources, date of honey extraction (day 0), and size of the groups at each testing apiary.

The experiment was conducted in six Mediterranean countries in the summer of 2021 (Figure 1). A total of nine test apiaries and 178 honey bee colonies were involved in the study. The study protocol (Supplement File S1) involved selecting full-size colonies of similar comparative strength in each apiary and dividing them into three homogeneous groups.

**Figure 2:**
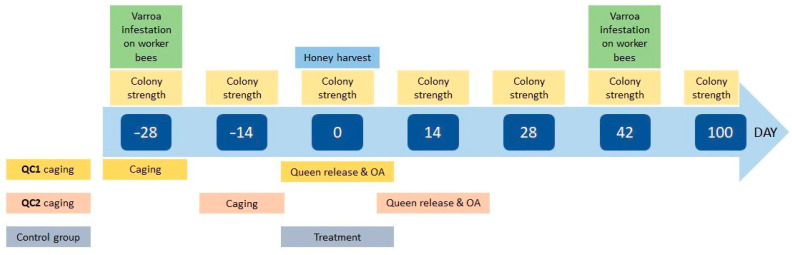
Timeline of the activities during the course of the study. Assessments listed above the timeline were performed on all colonies at the given day. Activities listed below the timeline were performed on a specific group at the given day.

In the first (QC1) and second (QC2) groups, queens were caged for a total of 28 days in a small-sized cage without the possibility of laying eggs [10] (Figure 2). Briefly, queens from the QC1 group were caged 28 days before “day 0” (day of the expected honey harvest of the main summer nectar flow), while queens from the QC2 group were caged 14 days prior to, and released 14 days after, “day 0”. In the control (C) group, queens were not caged. Honey bee colonies in QC1 and QC2 groups were treated, after queen release, on day 0 and day 14, respectively, by trickling 5 mL of oxalic acid 4.2% solution per occupied comb [10], while control colonies were treated using the usual local treatment (such as Apivar, Apiraz, CheckMite, formic acid, and total brood removal).

Colony strength was assessed by counting the number of combs occupied by adult bees and combs with brood, as previously described [34]. The net amount of honey produced by each colony was measured by weighing the honey super before and after extraction. The infestation rate of *V. destructor* on adult bees was determined using either the alcohol/soapy water wash or the powder sugar shake method [35]. The number of *V. destructor* mites per 100 bees was calculated following the method described by Dietemann et al., 2013 [36]. As a general rule, no major colony management techniques/methods that could potentially bias colony development and mite population growth were applied during the test period.

The timeline of the study activities is reported in Figure 2, where the timing of the queens’ caging, the frequency of honey bee colony strength estimations, the monitoring of *V. destructor* infestation, and the colony treatments are also shown.

Statistical analysis was performed in the SPSS software package, release 19.0 (SPSS Inc., Chicago, IL, USA). The effect of the fixed factors, namely, location (apiary, N = 9), study group (Q1, Q2, C), and their interaction (N = 16) on honey bee colony strength (number of combs occupied with bees and number of brood combs), were analyzed using a GLM ANOVA model. The same GLM model was applied to analyze honey production (only one measurement) and *V. destructor* infestation at the beginning and at the end of the experiment. Adjusted means between study groups were compared using Bonferroni post hoc analysis. Pearson’s correlation (r) analysis was used to calculate the correlation between colony strength and honey production.

## 3. Results

### 3.1. Colony Strength

**Figure 3:**
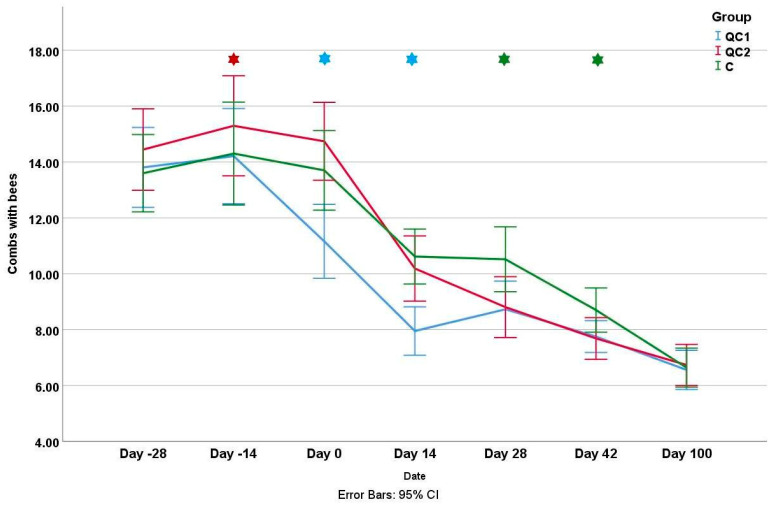
The mean number of combs occupied with bees during the experiment for three different groups. Stars represent significant differences between groups at a certain inspection day and the color of the star shows which group differs.

**Figure 4:**
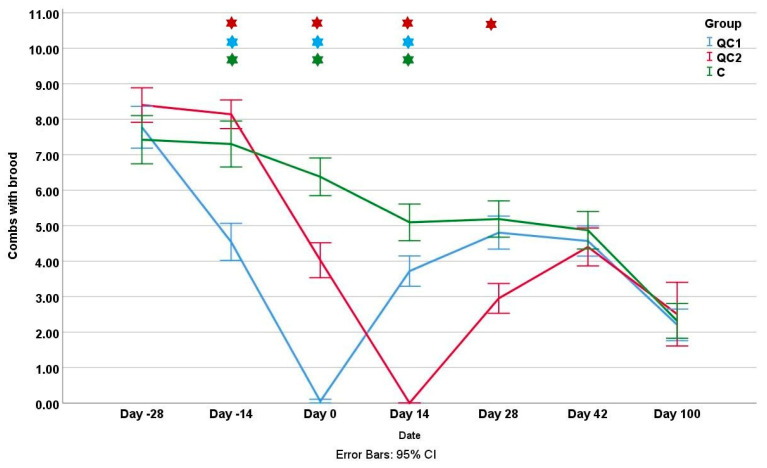
The mean number of brood combs during the experiment for three different groups. Stars represent significant differences between groups on a certain inspection day and the color of the star shows which group differs.

The apiaries involved differed significantly (*p* < 0.01) in colony strength, as assessed by the number of combs occupied with bees and the number of brood combs at the beginning (day −28) and end (day 100) of the study (Table 1 and Table 2). However, there were no significant differences in colony strength between the groups at the beginning and end of the study (*p* > 0.05, Figure 3 and Figure 4). On the inspection at “day −14”, the QC2 group had a significantly higher number of combs with bees compared to the other two groups (*p* < 0.01), while on the next two measurements (days 0 and 14), the QC1 group had a significantly lower number of combs with bees compared to the other two groups. On “day 28” and “day 42”, the colonies from the C group had significantly more combs occupied with bees compared to the other groups, as a consequence of queen caging.

In the next three measurements (day −14 to day 14), all groups differed significantly from each other (*p* < 0.01). On the “day 28” inspection, the QC2 group had significantly fewer brood combs compared to the other two groups (*p* < 0.01), while in the last two inspections, there were no significant differences in the amount of brood.

### 3.2. Honey Production

The honey yield differed significantly between the groups and apiaries (Table 3). The average amount of extracted honey per colony across all apiaries was 14.353 ± 0.429 kg (mean ± SD). Generally, the highest honey production was recorded in Israel (29.5 ± 7.15 kg), while the lowest was in Greece (8.19 ± 2.44 kg). Significantly lower amounts of honey were extracted from the QC1 group compared to both the QC2 (*p* = 0.001) and C groups (*p* = 0.009, Table 4. However, there was no significant difference between the QC2 and C groups. The initial colony strength had a significant effect on production. Pearson’s correlation analysis revealed a significant moderate positive correlation between honey extraction and the number of combs occupied with bees (r = 0.629, *p* < 0.01) as well as the number of brood combs (r = 0.257, *p* < 0.01) on “day −28” at the beginning of the study.

### 3.3. V. destructor Infestation

At the beginning of the field study, the infestation of adult bees with *V. destructor* varied between apiaries but not between groups (Table 5). On “day −28”, the infestation of adult bees (mean ± standard error) in the QC1, QC2, and C groups was 1.64 ± 0.19, 1.48 ± 0.22, and 1.52 ± 0.19 mites per 100 bees, respectively. However, on day 42 (following the completion of the control group treatment), there were significant differences in the infestation rate both between apiaries and between the groups. Specifically, the infestation rates were reduced to 0.44 ± 0.13 (QC1), 0.63 ± 0.16 (QC2), and 1.18 ± 0.13 (C) mites per 100 bees, with the C group exhibiting a significantly higher infestation compared to the QC1 group (*p* < 0.01) and QC2 group (*p* = 0.003).

## 4. Discussion

Here, we studied the impact of caging the queen at different times during the main summer nectar flow in combination with an oxalic acid treatment on honey production, *V. destructor* population, and honey bee colony development until winter. Our data support the usage of queen caging to achieve artificial summer brood interruption and the following oxalic acid application as a strategy for efficient *V. destructor* control. The number of adult bees in the autumn is negatively correlated to the *V. destructor* infestation level in the previous summer. Further, the number of bees in spring is negatively correlated to the *V. destructor* infestation levels in the previous October [37,38], so an efficient method of treating against Varroosis during the summer would improve the adult bee population needed for overwintering. We found that the timing of queen caging played an important role in the subsequent productivity of the colonies. Honey bee colonies in which queens were caged at the beginning of the summer nectar flow (QC2 group) produced, on average, the same amount of honey as the control colonies (where queens were not caged), while colonies in the QC1 group produced significantly less (on average 3–4 kg or 20–25%). On the other hand, both caging groups had significantly lower mite infestation at the end of the experiment compared to the control group, thus demonstrating the high efficacy of the caging method. It should be noted that we only measured the harvested honey regardless of the honey stores in the brood chamber. During the period of brood interruption, colonies usually store part of their honey in the brood chamber, which will afterward be used for new brood development and may reduce the need for extra feeding.

The strength of the colonies in different groups at the beginning of the study was equal, as well as at the end, before the winter. Still, there is an obvious positive correlation between colony strength and honey production. This indicates that beekeepers need to closely monitor and maximize honey bee colony strength, particularly the adult bee population, prior to the honey flow. Even if this is a trivial recommendation, one should keep in mind the significant differences between the regions and climates and the recommendations provided by the literature for the particular region. Thus, our results show that the timing is equally as relevant as the method. One of the effects that may be expected following the brood break is that after a few weeks, young bees have low juvenile hormone titers [39] and high protein and vitellogenin concentrations [33], as in long-lived wintering bees, and live significantly longer [1,31]. After the queen is caged, the amount of brood that needs to be fed decreases, so young bees can reach higher longevity and may start foraging earlier [40,41]. This can at least partly explain why the caging groups reached the same wintering colony size as the control colonies, although those had a higher overall brood production.

The starting point in combating *V. destructor* should consider the effect of management strategy on honey production, as this is the hive product of greatest interest for most beekeepers. For instance, brood breaks resulting from swarming negatively affect mite population development [37,42] but also honey production [28,43]. Therefore, our first point of interest was how a different starting time of queen caging in relation to the beginning of the main summer nectar flow would affect the amount of the extracted honey. In our study, honey production was highest in the QC2 and control groups, showing that caging the queens two weeks before the start of the main summer nectar flow (group QC1) is too early. A possible reason for lower nectar intake is that the strength of these colonies dwindled when the summer flow started. In addition, the lower amount of brood pheromones may have a negative impact on nectar intake [44]. Colonies in which queens were caged at the beginning of the nectar flow (QC2) were as productive as colonies from the control group. Decreased number of bees after the honey harvest in caging groups was no longer so important from the beekeepers’ aspect because the strength of the colonies at day 100 was equal. However, caution should be taken when using this queen caging method, and adaptation to the local environment is recommended as differences occur in the duration of brooding and nectar flow among the different geographic regions [38]. If there is late summer or fall nectar flow expected, the question is how this would affect possible additional honey harvest, as the tested group of colonies reached the control colonies in strength before winter. In addition, once the queen is released from the cage into a crowded hive, she starts to lay intensively, and the resulting few frames of open brood might lead some foragers to revert to nurse bees [45]. However, we did not measure the strength of colonies from day 42 until day 100, and we did not distinguish when in this period colonies equalized in strength. Similar values were obtained by Kovačić et al. [27], where colonies with caged queens had a 20–35% bee population reduction 28 days after queen release. In the work of Lodesani et al. [31], equalization between caging and control groups happened at least 67 days after queen release, which corresponds to the three weeks after day 42 in our study (three complete brood cycles instead of two). Brood interruption by queen caging in September seems to be late, as it affects the strength of the colonies entering the winter [46]. On the other hand, early spring queen caging is shown to be effective in reducing mite load without a negative effect on honey production and final colony size when caging is performed 9 weeks before the main spring nectar flow [31].

At the beginning of the study, there were no significant differences in the infestation rate of *V. destructor* of the colonies from different groups. However, upon measurement of the infestation rate after the treatment, we found a significantly lower infestation rate in caging groups compared to the control group. The control group of the study was treated as “business as usual” and consisted of different well-known and verified methods by partners. However, in this testing season, brood interruption followed by the OA treatment was shown to be more effective. This confirms the results of the previous study [27], where caging groups also had higher efficacy. In this study, we used 4.2% oxalic acid solution, which is proven to be effective [10], and the correct concentration and dosage of treatment are two of the most important details which should be considered, as lower concentrations when using the trickling method [10] or lower quantity when using sublimation [18,46] will result in lower efficacy. It is essential to highlight that the brood break is also an effective control method for *Tropilaelaps* spp. mite [47], a new possible threat to the European beekeeping industry [48]. From an economic point of view, it is possible to reduce costs since low *V. destructor* infestation leads to lower cost requirements for treatments, higher quality products, vital colonies, higher survival rates, and fewer winter colony losses which, according to Popovska Stojanov et al. [49], has substantial economic negative consequences on the overall beekeeping operation.

It is essential to emphasize the advantages of the tested method from the aspect of food safety as this approach does not compromise honey or other products in the hive as oxalic acid does not leave residues. One of the main challenges for successful *V. destructor* management is to reduce the infestation level in time before the development of long-living winter bees starts. While most registered chemical products may not be applied before the last honey harvest, which is often too late in the season, brood interruption can be started some weeks earlier without adverse effects on honey production and the in-hive products’ safety. Given the growing reports of resistance of mites to the active substances of medicines [7,8,50,51] and the negative effects of pesticides residues in wax on drone semen viability [52] and on workers longevity [53], future strategies of colonies protection should mainly focus on biotechnical methods and breeding honey bees with increased resistance against *V. destructor* mites [54,55,56].

## Figures and Tables

**Table 1:** GLM analysis on colony strength (number of combs with bees and combs with brood) at the start of the experiment (at day −28), with apiary, group, and their interaction as fixed factors.

	Number of Combs with Bees			Number of Brood Combs		
Source	df	Mean Square	F	df	Mean Square	F
Model	26	1504.739	359.315 **	26	447.303	260.864 **
Apiary	8	570.201	136.158 **	8	80.272	46.814 **
Group	2	1.172	0.411	2	2.017	1.176
Apiary × Group	15	3.020	0.721	15	3.263	1.903 *
Error	152	4.188		152	1.715	
Total	178			178		
	R^2^ = 0.984 (adjusted R^2^ = 0.981)			R^2^ = 0.978 (adjusted R^2^ = 0.974)		

** *p* < 0.01; * *p* < 0.05 Bonferroni test.

**Table 2:** GLM analysis on colony strength (number of combs with bees and combs with brood) at the end of the experiment (at day 100), with apiary, group, and their interaction as fixed factors.

	Number of Combs with Bees			Number of Brood Combs		
Source	df	Mean Square	F	df	Mean Square	F
Model	26	297.319	123.159 **	26	43.323	12.773 **
Apiary	8	84.157	34.976 **	8	29.556	8.714 **
Group	2	0.538	0.224	2	2.786	0.821
Apiary × Group	15	2.978	1.237	15	1.805	0.532
Error	133	2.406		133	3.392	
Total	159			159		
	R^2^ = 0.960 (adjusted R^2^ = 0.952)			R^2^ = 0.714 (adjusted R^2^ = 0.658)		

** *p* < 0.01; Bonferroni test.

**Table 3:** GLM analysis for honey production. Apiary, group, and their interaction are set as fixed effects.

Source of Variation	df	Mean Square	F
Model	26	2087.885	67.980 **
Apiary	8	2146.907	69.901 **
Group	2	199.852	6.507 **
Apiary ×Group	15	80.000	2.605 **
Error	151	30.713	
Total	177		

R^2^ = 0.921 (adjusted R^2^ = 0.908); ** significance < 0.01; (** *p* < 0.01; Bonferroni test).

**Table 4:** Estimated marginal means of honey extraction (in kg) for different groups.

Group	Mean	Standard Error	95% Confidence Interval	
			Lower Bound	Upper Bound
QC1	12.091 ^a^	0.729	10.650	13.532
QC2	16.009 ^b^	0.803	14.423	17.596
C	15.142 ^b^	0.705	13.749	16.535

^a,b^ Different apex letters represent significant differences between the groups (adjustment for multiple comparisons: Bonferroni, *p* < 0.05).

**Table 5:** GLM analysis on the infestation of colonies with *V. destructor* at the beginning (day −28) and end of the experiment (day 42) with apiary and group as fixed factors.

	Infestation with *V. destructor* on day −28			Infestation with *V. destructor* on day 42		
Source	df	Mean Square	F	df	Mean Square	F
Model	11	61.277	25.277 **	11	15.185	15.186 **
Apiary	8	30.962	12.772 **	8	5.170	5.171 **
Group	2	0.417	0.172	2	8.894	8.894 **
Error	167	2.424		152	1.000	
Total	178			163		
	R^2^ = 0.625 (adjusted R^2^= 0.600)			R^2^ = 0.542 (adjusted R^2^= 0.511)		

** *p* < 0.01; Bonferroni test.

## Data Availability

The datasets analyzed during the present study are available upon reasonable request from the corresponding authors.

## Supplementary Materials

The following supporting information can be downloaded at: https://www.mdpi.com/article/10.3390/insects14090751/s1, Supplement file S1: Coloss_protocol_2021.
