# Supplementary material for: Honey vs. Mite—A Trade-Off Strategy by Applying Summer Brood Interruption for Varroa destructor Control in the Mediterranean Region
Source: Insects. 2023 Sep 7;14(9):751. doi: 10.3390/insects14090751 (PMC10531922; doi:10.3390/insects14090751)
Supplement: Supplementary file 1 [file insects-14-00751-s001.zip › insects-2563637-supplementary.pdf]

COLOSS VARROA TASKFORCE, BROOD INTERRUPTION STUDY

QUEEN CAGING WITH EMPHASIS ON HONEY PRODUCTION

Marin Kovačić[1], Aleksandar Uzunov[2,3], Zlatko Puškadija[1], Ralph Büchler[3]

[1]Josip Juraj Strossmayer University of Osijek, Faculty of Agrobiotechnical Sciences, Osijek, Croatia

[2]Ss. Cyril and Methodius University in Skopje, Faculty of Agricultural Sciences and Food, Skopje, Republic of Macedonia

[3]LLH, Bee Institute, Kirchhain, Germany

INTRODUCTION

Experience gained from seasonal brood interruption studies showed that queen caging with OA solution trickling afterwards presents an effective method to reduce varroa infestation level of colonies without negative impact on colony. However, the question of ideal timing of queen caging still stay elusive and differ between regions. The main questions that were raised during Varroa taskforce spring workshop in Tel-Aviv were: a) how queen caging affects the honey production in quantity and quality, b) what is the right time for queen caging in respect to a maximal summer honey harvest and c) how caging affects queens' survivability during and after the caging. Opposite experience presented by members of group highlighted the necessity of further experiments. The basic hypothesis is that with the timely caging of the queen, additional worker bees would be available for nectar intake and consequently the honey production will be greater. On the other hand, the importance of the brood presence and brood pheromones to colony's agility and performance is not completely understood. If the hypothesis is correct, this would give additional motivation to beekeepers in accepting brood interruption as an integral part of beekeeping.

To shed the light on these questions, the common experiment was proposed to take place during summer of 2019 on international scale. Each participating partner will test two groups of eight colonies (groups differ in timing of queen caging) and compare it to control "standard local" group.

EXPERIMENTAL DESIGN

Material and methods

- Apiary with 24 full size naturally infested colonies (or at least 15 colonies) of similar strength and infestation
- Var-Control Cage Mozzato (API-MO.BRU) varroa control cages
- 4.2 % oxalic acid
- veterinary medicinal product for treatment of control (local standard) group
- suggestion is to have one electronic scale on apiary placed under control, average strength colony
    - If there is no thermometer in electronic scale, one should be provided on apiary

Before the start of experiment, colonies of similar strength are chosen and divided into 3 comparable groups. The location of the testing apiary must promise a good summer nectar flow. The arrangement of colonies on apiary should minimize drifting (sufficient space between stands and orientation of hives in different directions).

From the previous field experience and dates of spring flows, it is necessary to define the expected date of summer honey harvest. This date will be marked as “day 0” in protocol (see calendar of activities below in Table 1). The real date of harvest may of course be adapted to the real flow and weather conditions but should in any case be realized between day -7 to 7. When it is determined, 28 days before day “0” the queens from first testing group are caged. The queens from second group are caged exactly 14 days after the first group, regardless the blooming period or weather conditions. The accurate activities that need to be performed per day according to protocol are defined in Table 1. The proper method of queen caging and OA treatment is described in “Seasonal brood interruption study 2017/2018” protocol (Büchler and Nanetti, 2017.). The treatment against varroa mite in control group is performed on usual, standard local method.

Bee population and brood area

Adult bee population is estimated with the number of combs occupied with bees seen from above and, if applicable, also from below. One comb of bees means that at least two thirds of comb is occupied by bees. Intermediate values (0.5) can be used to describe slight differences between colonies. As weather conditions may have effect on bee clustering, any unfavourable weather conditions during colony inspection should be noted. The estimation of the number of combs occupied by bees is made from above. The estimation is made with the limited use of smoke and without moving the combs.

The brood area is estimated with the number of combs with brood. All combs containing at least a small number of brood cells (size of tennis ball), independent from the stage of brood (eggs, larvae, pupae) are considered as a brood combs. Brood on only one side of the comb is marked as 0,5 comb of brood.

Honey harvest (net weight and water content)

For the purposes of this research, it is important to determine the net weight of honey production from the main summer flow. To achieve this, all supers (or frames) that are extracted should be weighted. At the same time, the water content of at least 5 randomly taken honey samples per group should be measured with a refractometer.

Adult bee infestation

The sample of 100 ml od adult bees is taken from the honey box. The infestation rate of bees is determined either with soapy water wash or powder sugar shake method. In both methods, it is necessary to weight the bee sample in order to get the accurate rate of bee infestation. It is calculated as the number of mites per 10 g of bees.

Database

Database will be shared among partners to collect all relevant data for a common evaluation of the experiment.

Feeding of colonies

The common practice in the region is followed for the stimulative feeding of colonies. If feeding of colonies after the caging is performed, all colonies should be fed on the same way and with the same amount.

Table 1. Calendar of activities

Your date	Day before expected / post honey harvest	Group 1	Group 2	Control
	**-28**	Colony strength<br>Bee infestation<br>Queen caging	Colony strength<br>Bee infestation	Colony strength<br>Bee infestation
	**-14**	Colony strength	Colony strength<br>Queen caging	Colony strength
	**0**	Colony strength<br>**Honey harvest**<br>Queen release<br>OA treatment	Colony strength<br>**Honey harvest**	Colony strength<br>**Honey harvest**<br>Standard treatment
	**14**	Colony strength	Colony strength<br>Queen release<br>OA treatment	Colony strength
	**28**	Colony strength	Colony strength	Colony strength
	**42**	Colony strength<br>Bee infestation	Colony strength<br>Bee infestation	Colony strength<br>Bee infestation
	**100**	Colony strength	Colony strength	Colony strength
